# Changes in Parkinson’s disease sleep symptoms and daytime somnolence after bilateral subthalamic deep brain stimulation in Parkinson’s disease

**DOI:** 10.1038/s41531-018-0053-5

**Published:** 2018-05-25

**Authors:** Siddharth Kharkar, Jonathan Richard Ellenbogen, Michael Samuel, Alexandra Rizos, Monty Silverdale, K Ray Chaudhuri, Keyoumars Ashkan

**Affiliations:** 1 Department of Neurology, Wockhardt Hospitals, Mumbai, Maharashtra India; 2 Department of Neurosurgery, King’s College Hospital NHS Foundation Trust, London, UK; 3 Department of Neurology, King’s College Hospital NHS Foundation Trust, London, UK; 4 EUROPAR Offices, King’s College Hospital NHS Foundation Trust, London, UK; 5 Department of Neurology, Salford Royal NHS Foundation Trust, Manchester, UK

## Abstract

Introduction: Deep brain stimulation (DBS) markedly improves motor function in advanced Parkinson’s disease (PD), but its effect on sleep is less clear. Patients and methods: Forty PD patients who had subthalamic DBS (STN-DBS) were identified from an on-going non-motor naturalistic longitudinal study (NILS). All patients were followed up for at least 6 months, 26 patients had a 1 year follow-up. A total PDSS score of 100 or less, a score in any PDSS-item of 6 or less, and a Epworth score of 10 or more were classified as being significant. Results: Forty-five percent of patients reported significant improvement in the total PDSS score at 6 months, and 35% at 12 months. In terms of magnitude, the total PDSS score at 6 months was significantly improved from baseline while the improvement at 12 months was not statistically significant. The most frequently reported improvements were overall sleep quality and maintenance of sleep. Some patients reported worsening of the total PDSS score. More than half of the patients reporting daytime sleepiness at baseline had persistent sleepiness at 6 and 12 months. The mean Epworth Score did not improve because a significant number of patients *without* sleepiness at baseline reported new-onset sleepiness at 6 and 12 months. Neither medication changes nor motor improvement were consistently related to sleep changes after DBS. Conclusion: Subthalamic DBS is associated with a statistically and clinically significant, but variable, improvement in sleep as measured by the PDSS. The most frequent improvements were better overall sleep quality and better sleep maintenance.

## Deep brain stimulation: variable effects on sleep

Subthalamic deep brain stimulation (DBS) improves sleep quality and maintenance in a subset of patients with Parkinson’s disease (PD). DBS is known to markedly improve motor function PD patients, but its effects on sleep are unclear. Most patients with advanced PD experience sleep problems that adversely affect their quality of life. Jonathan Ellenbogen, at Kings College Hospital in London, UK, and colleagues investigated the long-term effects of DBS on sleep in 40 patients using a PD-specific sleep symptom questionnaire. Eighteen patients (45%) reported a significant overall improvement in sleep quality and maintenance after 6 months, but three of them (7.5%) reported a worsening of sleep symptoms. There was no correlation between the improvement in motor function and sleep improvement following DBS indicating that further research is required to optimize the effects of DBS on sleep.

## Introduction

The high prevalence and marked impact of non-motor symptoms of Parkinson’s disease (PD) on quality of life^1,2^ are often underappreciated by treating physicians.^3,4^ Sleep problems are an integral aspect of the NMS of PD and are common in PD patients, and become increasingly more frequent and severe in advanced disease.^5,6^ Studies have reported that more than 95% of advanced PD patients have problems with sleep^7^ while recent studies in early PD have indicated an overall prevalence of about 49%.^6^ Practically any aspect of sleep may be affected, but the most consistently reported sleep problems in untreated PD are poor maintenance of sleep and sleep disturbed by nocturia.^8,9^

Dopaminergic therapy can improve aspects of sleep in PD patients. Long acting agents or short duration preparations given continuously (such as Apomorphine infusions) are particularly beneficial but even these strategies may not alleviate sleep symptoms completely.^10–12^ Conventional treatments for sleep problems, such as eszopiclone,^13^ clonazepam,^14^ and doxepin^15^ may improve sleep in PD patients; but need to be used carefully since they have the potential to worsen daytime sleepiness, cognition and balance in PD patients.

Deep brain stimulation (DBS) is an attractive treatment option for patients where advanced therapies are indicated.^16^ While DBS markedly improves motor functioning^17,18^ its effect on sleep is less clear. There are only a handful of studies which utilize PD-specific sleep scales for evaluation of sleep changes after DBS, and some of these are compromised by small sample sizes and short duration of post-surgery follow-up.

We surveyed long term changes in subjective reporting of sleep symptoms (nocturnal and daytime) in 40 patients with Parkinson’s disease who had Subthalamic Deep Brain Stimulation (STN-DBS), using the PDSS and Epworth Sleep Scale. We conducted this study with the aim of documenting changes in sleep symptoms after STN-DBS. A secondary aim was to explore the influence of motor improvement and medication changes on sleep symptoms after DBS.

## Results

### Demographics and baseline characteristics

Patient demographics and baseline characteristics are presented in Table 1. A majority of patients (65%) had significant overall impairment of sleep (total score < = 100) at baseline. The most frequent sleep symptoms were bad overall sleep quality (PDSS-1: 75%), difficulty maintaining sleep (PDSS-3: 82.5%), and getting up to pass urine (PDSS-8: 80%). 40% (16/40) patients had significant daytime sleepiness at baseline as measured by the Epworth sleepiness scale.

**Table 1:** Demographics

	Mean (SD) or number (percentage)
Number of patients	
Baseline	40
6 month follow-up	40
1 year follow-up	26
Gender (Females)	15 37.5%)
Age at onset of symptoms	48.7 ± 7.7
Age at the time of DBS surgery	59.6 ± 8.3
Duration of symptoms before DBS surgery (years)	10.7 ± 4.5
PDSS-total ( < = 100)	26 (65%)
PDSS-1: Overall sleep quality	30 (75%)
PDSS-2: Difficulty falling asleep	15 (37.5%)
PDSS-3: Difficulty staying asleep	33 (82.5%)
PDSS-4: Restlessness of legs or arms	18 (45%)
PDSS-5: Fidgeting in bed	19 (47.5%)
PDSS-6: Distressing dreams	9 (22.5%)
PDSS-7: Distressing hallucinations at night	1 (2.5 %)
PDSS-8: Getting up to pass urine	32 (80%)
PDSS-9: Urinary incontinence due to immobility	4 (10%)
PDSS-10: Numbness or tingling of arms/legs	10 (25 %)
PDSS-11: Muscle cramps while sleeping	18 (45%)
PDSS-12: Painful posturing on waking	17 (42.5%)
PDSS-13: Tremor on waking	21 (52.5%)
PDSS-14: Tired and sleepy on waking	23 (57.5%)
PDSS-15: Unexpectedly falling asleep during the day	17 (42.5%)
Epworth sleepiness scale	16 (40%)

### Sleep changes after DBS

Forty-five percent (18/40) patients displayed significant improvement in the total PDSS score at 6 months, and 35% (9/26) at 12 months. In terms of magnitude, the total PDSS score improved at 6 months by 23.5% (from a median score of 91.5 to 113.5, *p* = 0.001). The median score at12 months (median = 102) was higher than that at baseline, but this difference was not statistically significant (*p* = 0.07).

**Fig. 1:**
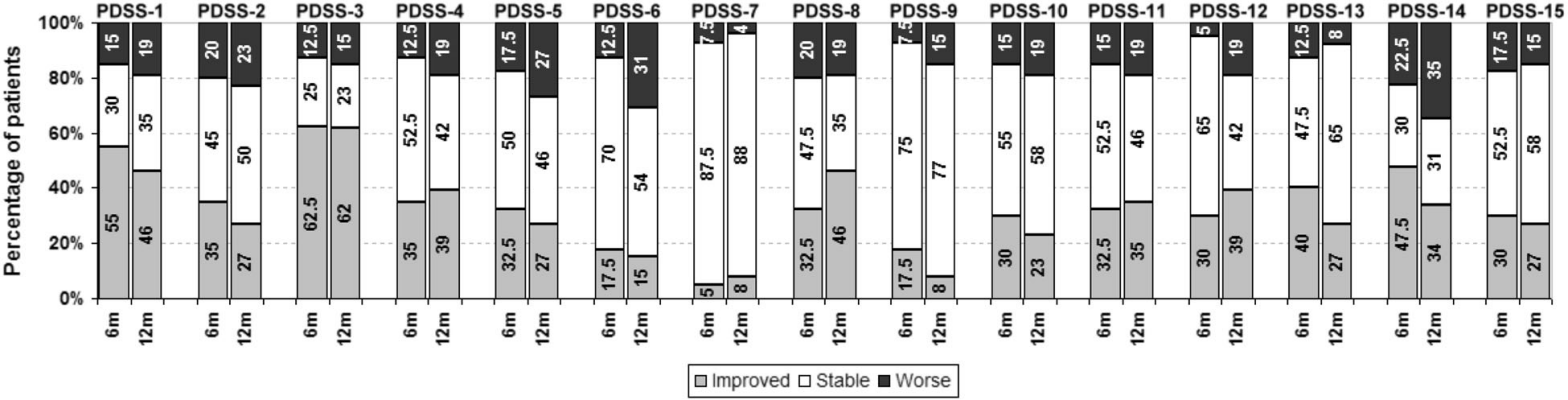
Percentage of patients reporting a change in PDSS sub-scores at 6 and 12 months

**Fig. 2:**
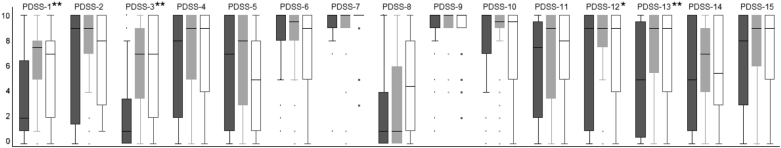
Median and range of PDSS sub-scores at baseline, 6 and 12 months. (*6 month score statistically different from baseline, **both 6 and 12 month scores statistically different from baseline)

The most frequently reported changes (Fig. 1) were improved overall sleep quality (PDSS-1: 55% and 46% of patients at 6 months and 12 months, respectively) and improved maintenance of sleep (PDSS-3: 62.5% and 62% of patients at 6 and 12 months). Patients also frequently reported improvement in tremor on waking (PDSS-13: 40% and 27% of patients at 6 and 12 months) and painful posturing of legs in the morning (PDSS-12: 30% and 38% of patients at 6 and 12 months). At 6 months, the median improvements in all of these aspects of sleep were statistically significant (Fig. 2). 1 year improvement showed a similar pattern, but only the improvements in overall sleep quality, sleep maintenance, and tremor on waking were statistically significant, although this may be because of small numbers who reached 1 year follow up.

7.5% (3/40) patients reported significant worsening of the total PDSS score at 6 months, and 19% (5/26) at 12 months. In addition, for each sleep symptom, some patients reported deterioration (Fig. 1). In particular, patients reported a very variable response in terms of feeling tired and sleepy after waking up (PDSS-14). Patients frequently reported feeling less tired and sleepy after waking (47 and 35% at 6 months and 12 months) but, a substantial number of other patients also reported being more tired and sleepy on awakening (22.5% at 6 months and 35% at 12 months) and hence there was no improvement in the PDSS-14 median score.

Of the 16 patients with significant daytime sleepiness at baseline as measured by the ESS, 56% (9/16) had persistent daytime sleepiness at 6 months and 81% (9/11) at 12 months. In terms of magnitude, the Epworth Score did not improve because a significant number of patients *without* sleepiness at baseline reported new-onset sleepiness at 6 months (33%, 8/24) and at 12 months (20%, 3/15).

There was no statistically significant difference in the percentage improvement in the PDSS total scores between patients who showed moderate improvement of UPDRS scores after levodopa challenge, as compared to those who showed marked improvement.

### Changes in medications after DBS

The mean LED dose decreased by 25% at 6 months (from 1051 ± 413 mg at baseline to 786 ± 340 mg) and by 30.5% (to 730 ± 417 mg) at the 1 year follow-up. We did not detect an association between decrease of dopaminergic medications and changes in the total PDSS score. At 6 months, larger decreases in total LEDD were weakly associated with a deterioration in the Epworth Sleepiness score (Rho: −0.31, *p* = 0.049). No such relationship was found at 12 months.

### Changes in motor scores after DBS

As expected, the SCOPA-motor score improved in most patients. The mean SCOPA-motor score improved by 35% at 6 months (from 25.3 ± 10.1 to 16.4 ± 8.8, *p* < 0.0001) and by 40% at 12 months (to 15.2 ± 8.15, *p* < 0.0001). No clear relationship was discernible between improvement in SCOPA-motor scores and global improvement of sleep (total PDSS score) at either 6 or 12 months (Spearman’s correlation).

## Discussion

In this study, subthalamic DBS was followed by a statistically and clinically significant, but variable, improvement in sleep as measured by the PDSS. The most frequent improvements were better overall sleep quality and better sleep maintenance. However, a variable but significant proportion of patients experienced deterioration in different aspects of sleep (Fig. 1). Sleep improvement after DBS did not correlate with improvement in UPDRS scores after levodopa challenge testing prior to DBS insertion, medication changes after DBS or motor improvement after DBS. At this point, we believe that it is reasonable to counsel patients that while different aspects of their sleep may improve or deteriorate after DBS; the most likely possibility is that they will have an overall improvement in their sleep.

The present study is the largest study utilizing a PD-specific sleep symptom questionnaire. A previous large study of sleep symptoms in PD after *unilateral* STN-DBS did not utilize a PD-specific sleep questionnaire, although the results reported were comparable to the present study.^19^ Polysomnography (PSG) studies of PD patients after DBS have documented a reduction in sleep disturbance, decrease in wakefulness after sleep onset, improvement in sleep efficiency and total sleep time, and increased REM sleep.^20–23^ These PSG changes are compatible with the changes documented in our study.

There is considerable variation in the results of previous studies utilizing the PDSS on post-DBS sleep changes^24–27^ (Table 2). The most consistent and significant change is seen in sleep maintenance (PDSS-3), in keeping with our study. It is possible that patients interpret this change as an improvement in their overall sleep quality (PDSS-1). In addition, we also noted substantial improvement in tremor and painful posturing after waking (PDSS-12 and 13), which is likely related to better control of motor symptoms by DBS even before the morning dose of dopaminergic medication is ingested.

**Table 2:** Studies utilizing Parkinson’s specific sleep measures for the evaluation of post-DBS sleep changes:

Study	Number of patients, surgery	Follow-up after surgery	Sleep evaluation instruments	Overall effect on sleep	Effect on onset and maintenance insomnia	Effect on motor symptoms	Effect on REM, RBD and RLS/PLMS	Effect on sleep refreshment and daytime sleepiness	Mean reduction Dopamine agonist dosage	Mean reduction in levodopa dosage	Mean reduction in total dopaminergic dosage
Deli 2015^31^	25 bilateral STN	Single follow-up at 12 months	PDSS-2, Epworth sleepiness scale	Total PDSS score improved by 43%	No significant change	Reduction in immobility at night, muscle cramps and tremor in the morning	Reduction in restlessness of legs/arms	Reduction in daytime sleepiness	18%	63%	43%
Chahine 2011^32^	5 bilateral, 12 unilateral STN	Multiple follow-ups upto 6 months	PDSS, Epworth sleepiness scale, IRLSSG rating scale	Total PDSS score improved by 33%	Reduced difficulty in staying asleep	No significant change on PDSS	No significant change in RLS symptoms. No de-novo RLS cases	Reduction in daytime sleepiness	Not specified	Not specified	50%
Nishida 2011^33^	8 bilateral, 2 unilateral STN	Single follow-up 1 week after programming	PDSS, polysomnography	Total PDSS score improved by 20%	Strikingly decreased wakefulness after sleep onset on polysomnography (58% reduction)	Not specified	-1 out of 2 patients with dream enactment and nocturnal vocalizations improved-Two patients developed de-novo dream enactment behaviors-REM sleep with atonia (normalREM) increased, slow wavesleep increased particularly in thefirst half of the night	Reduction in day time sleepiness	–	Not specified	Not specified
Hjort 2004^34^	10 bilateral STN	Single follow-up at 3 months	PDSS	Total PDSS score improved by 32%	Reduced difficulty in falling asleep	Decreased restlessness of arms, painful cramps, painful posturing and tremor on waking	No significant change	No significant change	Not specified	Not specified	29%

Previous research has suggested that a marked decrease in dopaminergic medications (by 79% or more) after STN-DBS may worsen or lead to the emergence of restless legs syndrome and REM behavior disorder.^28,29^ In this study, a significant number of patients developed worsening restlessness of legs, distressing dreams and hallucinations at night (Fig. 2: PDSS-4, PDSS-6, and PDSS-7) even though the decrease in dopaminergic medications was modest. There is uncertainty regarding the relationship between leg motor restlessness in PD and true RLS,^30,31^ and polysomnography is required for the definitive diagnosis of RBD.^32^ Hence, these findings should be interpreted with caution.

The PDSS assesses nocturnal problems, sleep disturbances and excessive daytime sleepiness and is composed of 15 items, addressing nocturnal symptoms commonly associated with PD (insomnia, nocturia, nocturnal motor symptoms, etc.). Each item is rated on a visual analog scale (VAS) from 0 (severe or always present) to 10 (never or not present). The total score is obtained by summing the items. The time frame is the previous week. It is specific for PD, is extensively used and validated and has been shown to be responsive to changes and is recommended by the MDS Task Force.^33^ Studies have also suggested data from PDSS correlate with PSG based datasets and worldwide reports from studies using PDSS indicate that scores below PDSS-total of 100 indicate abnormal sleep. The PDSS is useful to identify PD patients who will have abnormal PSG recordings,^34^ but the correlation between subjective and objective measures of sleep can be imperfect.^35^ We believe that subjective and objective measures of sleep are complimentary; neither one is necessarily “superior” to the other. Although it is clear that subjective sleep assessments are fundamental to complaints of insomnia and non-restorative sleep, two conditions associated with considerable morbidity and impairment in PD.^36^

Arnulf et al.^20^ found that night-time awakenings, a normal phenomenon, were often followed by dystonia and extended awakenings. They hypothesized that the continuous nature of DBS stimulation may provide better motor control at night, preventing such extended awakenings. Our univariate analyses indicated that improvement in motor function was not *sufficient* for an improvement in sleep maintenance; however its contribution needs to be evaluated with multivariate analysis in a larger sample. We detected an increase in daytime sleepiness associated with reduction of high doses of total LEDD. This is possibly the result of falling in the trough of the sleep-response to levodopa, wherein more moderate doses of levodopa are associated with sleepiness and higher doses with better wakefulness, as described by Bliwise et al.^37^

In addition to motor improvement and medication changes, multiple other factors may affect post-DBS sleep, including changes in depressive symptoms^38^ and possibly a direct effect of DBS on sleep structures. It is a difficult task to assess the relative contributions of these factors. GPi-DBS is usually followed by smaller decrease in dopaminergic medication^39^and it may be possible to gain a better understanding of the relative importance of motor improvement without this additional confounder. In a randomized trial of GPi-DBS versus STN-DBS,^39^ the magnitude of sleep improvement measured by PDSS was similar in both groups, possibly indicating a durable effect common to both GPi and STN DBS that surpasses any effect of dopaminergic medication withdrawal.

There are limitations to our study. First, we did not assess sleep architecture formally, as discussed above. However, the globally validated PDSS use does offset the lack of PSG and additionally allows inclusion of motor and other PD symptoms which affect sleep. Second, we did not assess the effect of depression. Changes in mood which may occur in some patients after STN-DBS^38^ could have worsened sleep symptoms^40^ and diluted our results. Third, we did not undertake multivariate analysis. In our view, multivariate analyses could be misleading given the relatively small patient population. Since this was a preliminary study, we did not use a multiple comparison correction such as Bonferroni, which can substantially increase the chance of a type II error.^41^ Finally, the cut-offs we used for defining significant change in sleep symptoms need to be prospectively validated.

In conclusion, our study confirms improvement in some sleep symptoms after STN-DBS, in particular overall sleep quality and sleep maintenance. Our data indicates that improvement in motor function is not sufficient for sleep improvement. Further studies need to focus on the reasons for these improvements with the goal of refining DBS and medication management strategies after STN-DBS to maximize sleep improvement.

## Methods

### Patients

40 patients who had STN-DBS for Parkinson’s disease and were enrolled in the on-going non-motor naturalistic longitudinal study (NILS, UK clinical research network number 10084). The NILS study received ethical approval from all local Research Ethics Committees at each centre, and all patients provided informed written consent for inclusion in the study. All patients were followed up for at least 6 months, 26 of these patients also had a 1 year follow-up.

All patients met British Brain Bank criteria^42^ for PD, and were selected for DBS due to insufficient control of their motor symptoms by medication. Pre-surgery response to levodopa was verified in all cases by a >30% improvement in the Unified Parkinson’s Disease Rating Scale (UPDRS)-III motor score after a standard levodopa challenge dose. None of the patients had significant psychiatric illnesses or dementia which would preclude DBS implantation. All patients had pre-operative full multidisciplinary assessment by our dedicated DBS team, which includes a neuropsychologist and a neuropsychiatrist.

All patients in this study had bilateral subthalamic implantation of DBS electrodes, followed by initiation of stimulation within 6 weeks. DBS programmer settings were changed per clinical requirements at the discretion of the treating physician / DBS specialist nurse. Patients were evaluated at fixed intervals per the NILS protocol: within one month before DBS surgery, 6 months after surgery, and yearly thereafter. All 40 patients had a baseline and 6 month evaluation. At the present time, 26 patients have had the 12 month evaluation.

### Measurement instruments

1. Sleep symptoms were assessed by the Parkinson’s Disease Sleep Scale (PDSS),^36^ a patient reported 15 item scale with each item weighed from 0 to 10. Lower scores on each item represent worse symptoms: 0 indicates that the patient “always” has the symptom and 10 represents an answer of “never”. Correspondingly, the total PDSS score varies between 0 (worst) and 150 (best).
2. Daytime sleepiness was assessed using the Epworth Sleepiness Scale,^43^ which is an 8-item scale that assesses the likelihood of falling asleep while performing common tasks during the day. A score of 0 indicates no daytime sleepiness, while the maximum score of 24 indicates the highest level of daytime sleepiness in all tested situations.
3. Motor improvement was measured by the motor subpart of the SCOPA scale (SCOPA-motor),^44^ a 21 item scale which correlates strongly with the corresponding subpart of UPDRS-III.^44,45^ Higher scores on SCOPA-motor represent worse motor symptoms, the maximum score is 75.

### Categorization of scores

Patients with a total PDSS score of 100 or less,^10^ a score in any PDSS-item of 6 or less, and a Epworth score of 10 or more^46^ were classified as having significant problems. A change of the total PDSS score by 20 points or more, or in one of the sub-scores by 2 points or more was considered clinically significant. Follow-up scores were classified as improved, stable or worse as compared to the baseline scores. The criteria for defining clinically significant change in PDSS scores were based on our clinical experience and have not been formally validated. The percentage improvement in UPDRS score after levodopa challenge was categorized into two categories: “Moderate” if the improvement was between 30–65% and “Marked” if the improvement was 65% or above.

### Medication changes

The levodopa equivalent daily dose (LEDD) was computed according to the widely accepted method described by Tomlinson et al.^47^

### Statistical analysis

Data analysis was done in Stata 12 (Stata Corp, Texas). Normality was tested using the Shapiro-Wilk test. For pre-DBS and post-DBS comparisons, the Wilcoxon signed-rank test was used for comparing non-normally distributed variables and the paired student t-test was used for normally distributed variables, corresponding non-paired tests were used for other comparisons.

### Data availability statement

The data is maintained by Ms. Alexandra Rizos, Research Manager at Kings College Hospital, and can be made available for review upon request.
